# Ultrasound tracked motion compensated focused ultrasound system evaluated on e*x vivo* ovine livers

**DOI:** 10.1186/2050-5736-3-S1-P52

**Published:** 2015-06-30

**Authors:** Jan Strehlow, Xu Xiao, Michael Schwenke, Ioannis Karakitsios, Markus Domschke, Senay Mihcin, Yoav Levy, Tobias Preusser, Andreas Melzer

**Affiliations:** 1 Fraunhofer MEVIS, Bremen, Germany; 2 Institute for Medical Science and Technology, Dundee, United Kingdom; 3 University of Dundee, Dundee, United Kingdom; 4 InSightec Ltd, Tirat Carmel, Israel; 5 Fraunhofer MEVIS/Jacobs University Bremen, Bremen, Germany

## Background/introduction

The application of FUS in abdominal organs such as the liver or the kidneys is impeded by a number of complications. One of the most challenging is organ motion due to breathing. To achieve ablation in a target within a moving organ the FUS system has to be steered to focus on the same anatomical position. We present a prototypical system that tracks the motion of an *ex vivo* ovine liver via diagnostic ultrasound (US) and adjusts the focal spot to a fixed anatomical position.

## Methods

**Figure 1:**
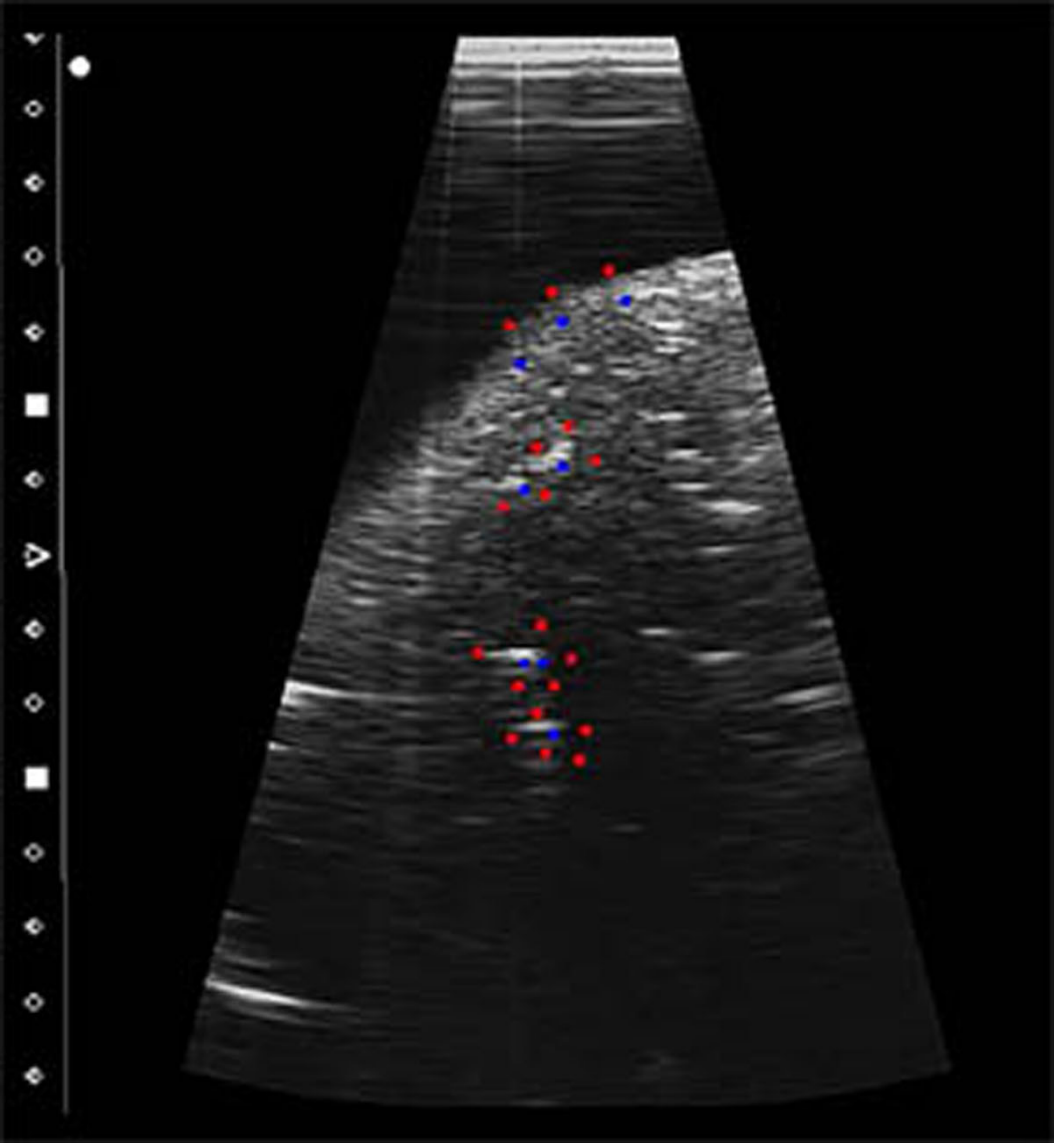
Liver vessels used as tracking features to assess organ motion

Our setup consists of four systems: An organ in breathing-like motion is modeled by a robotic arm periodically moving a fresh ovine liver in water tank. The liver motion is assumed to be unknown, its movement range is 20 mm, and one cycle takes approximately 8 seconds. A diagnostic US system is used to track vessels in the liver (Figure 1). Real time tracking positions are send to a therapy control system that calculates the position of a target using a linear motion model. The therapy control adjusts the focal spot of a steerable FUS system to match the computed target position. Since an active FUS will render our US-tracking images unusable the FUS is pulsed, leaving short imaging windows for tracking. Temperature is assessed by a thermocouple inserted into the liver. The target area is defined in vicinity of the thermocouple. Temperature curves of a moving and a static organ are acquired for different update rates and different output powers of the FUS system. The temperature curves of static and moving scenarios are compared to assess the ability of the system to compensate motion.

## Results and conclusions

**Figure 2:**
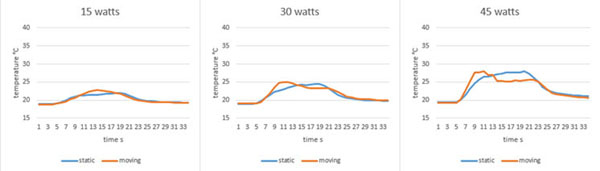
Comparison of temperature curves measured during sonications of static and moving organ. Focal spot position was updated with 9 Hz and tracking images were acquired every 3 Hz. Output power was a) 15 W, b) 30 W, and c) 45 W

The US-tracking of the organ works reliably when FUS was switched off for at least 80 ms with 2-4 Hz. The output power of the FUS system does not influence the reliability of the system. Temperature curves for the static and moving scenarios show only minor differences. Figure 2 shows the temperature curves acquired by sonication with a) 15 W, b) 30 W, and c) 45 W for 20 seconds with a sonication update rate of 9 Hz and 3 Hz US-tracking. The average temperature differences are a) 0.33 °C b) 0.56 °C, and c) 1.0 °C. The area under the curve differs by a) 14.09%, b) 1.17%, and c) 0.99% (static scenario as reference). Our US-tracked steered FUS system can compensates unknown motion that is similar to the one induced by respiration. In our phantom a linear motion model was sufficient to compute the target position. For *in vivo* experiments this motion model has to be changed to a deformable one. The system, however, demonstrates the feasibility of US tracked steered FUS.

